# Supplementary material for: PKC-Mediated Orai1 Channel Phosphorylation Modulates Ca2+ Signaling in HeLa Cells
Source: Cells. 2022 Jun 27;11(13):2037. doi: 10.3390/cells11132037 (PMC9266177; doi:10.3390/cells11132037)
Supplement: Supplementary file 1 [file cells-11-02037-s001.zip › cells-1662930-supplementary.pdf]

**Supplementary Information**

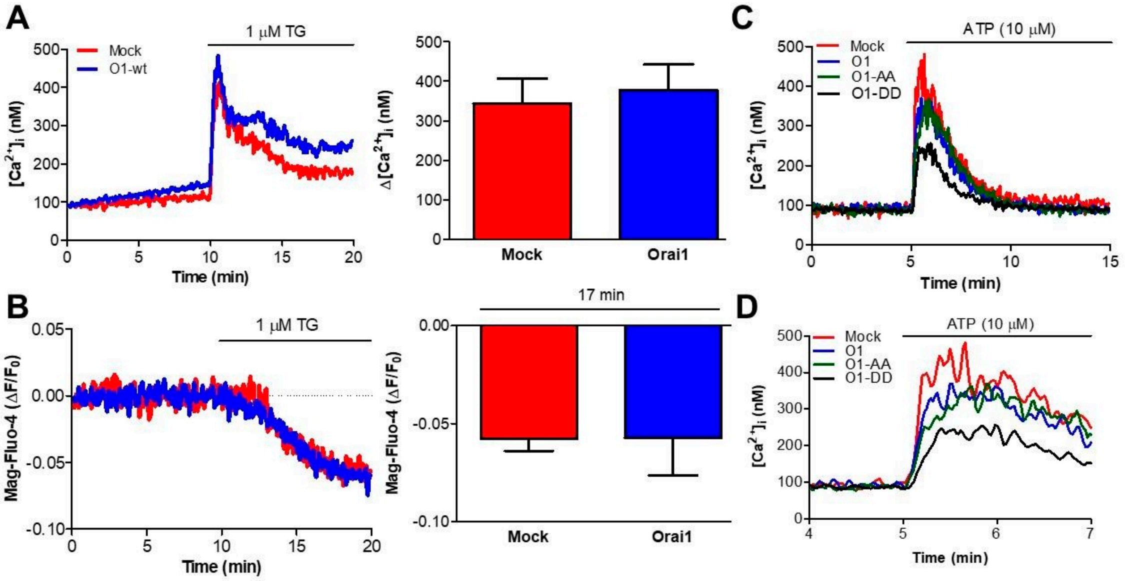


**Figure S1. The overexpression of O1-wt did not modify TG-evoked $Ca^{2+}$- responses but inhibited the agonist-induced intracellular $Ca^{2+}$ concentration ($[Ca^{2+}]_i$) response in conditions for limited $Ca^{2+}$ entry (high $K^+$ saline solution).** (**A**) Time course of $[Ca^{2+}]_i$ for cells recorded in saline solution and overexpressing O1-wt (blue trace) or transfected with an empty plasmid (mock, red trace), the peak $\Delta[Ca^{2+}]_i$ did not show any difference induced by 1 μM TG. (**B**) The concomitant time course of TG-evoked luminal $Ca^{2+}$ reduction showed a similar reduction in cells overexpressing O1-wt (blue trace) and cells transfected with an empty plasmid (red trace). Data show the mean ± SEM for n = 4**.** (**C**) ATP-induced $[Ca^{2+}]_i$ responses for HeLa cells resuspended in isotonic high $K^+$ saline solution (126.4 mM KCl, 0.8 mM $MgCl_2$, 6 mM $NaHCO_3$, 1.8 mM $CaCl_2$, 5.5 mM glucose, and 25 mM Hepes (pH 7.3 adjusted with NaOH) overexpressing O1-wt (blue trace), O1-AA (green trace) and O1-DD (black trace). Membrane depolarization strongly reduces $Ca^{2+}$ entry by plasma membrane $Ca^{2+}$ channels. (**D**) The initial 2-min $[Ca^{2+}]_i$ response from the panel C data.

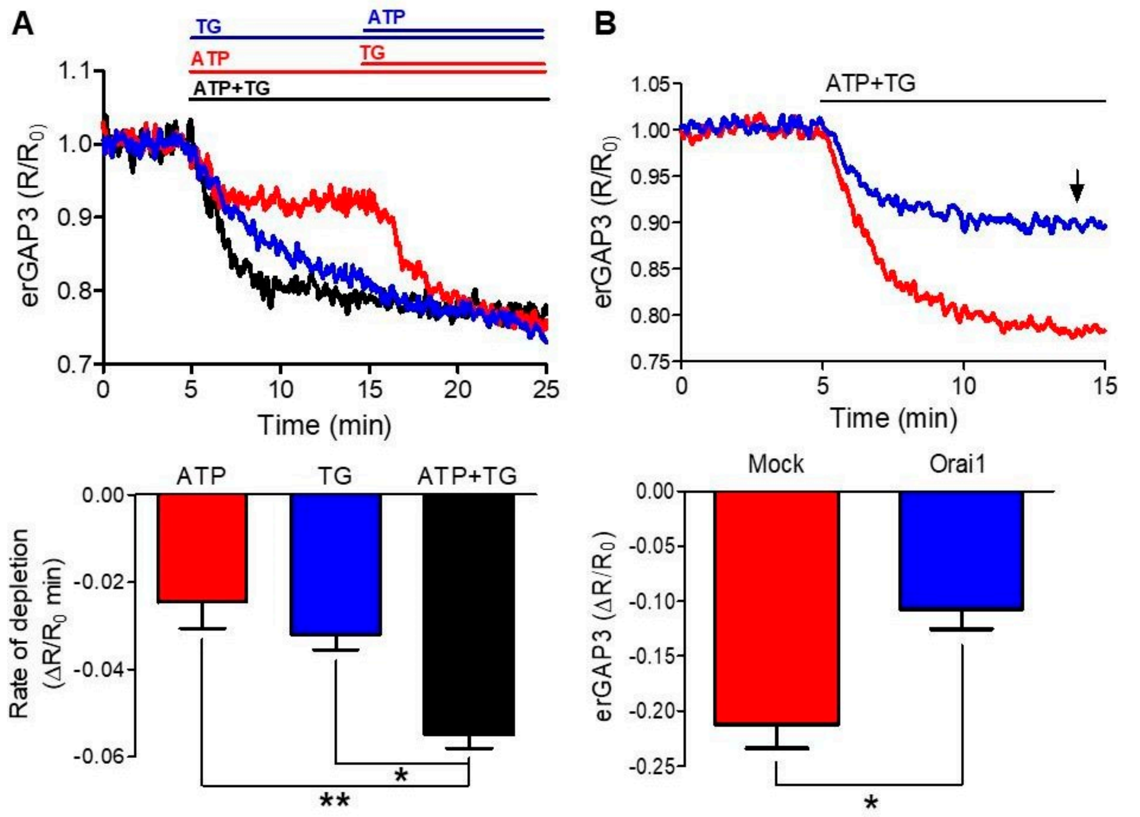


**Figure S2. The combination of ATP and TG rapidly depleted the ER $Ca^{2+}$ store, and the overexpression of O1-wt inhibited this depletion.** (**A**) HeLa cells were transfected with an ER-targeted Genetically Encoded $Ca^{2+}$ indicator (erGAP3) for 24 h. Traces show the ER [$Ca^{2+}$] responses in cells stimulated either with ATP and TG (black trace) or first ATP followed by TG after 10 min (red trace) or vice versa (blue trace). Applying together ATP and TG showed a faster $Ca^{2+}$ reduction from the ER. Data represent the mean ± SEM for n = 3. Statistical analysis was carried out with one-way ANOVA with Dunnett's correction for multiple comparisons using ATP and TG as the reference. (**B**) HeLa cells were co-transfected with the erGAP3 indicator with either Orai1 (black trace) or an empty plasmid (mock, red trace). The fluorescence ratio time course was assessed 24 hours later in response to 10 μM ATP and 1 μMTG applied at the indicated time (arrow). Fluorescence ratio changes are the average response of 1 × $10^6$ cells/ml in saline solution with 1.8 mM $CaCl_2$. The erGAP3 $Ca^{2+}$ indicator fluorescence was stimulated at 405 and 470 nm and collected at 515 nm. Notice that the presence of the O1-wt channel resulted in substantial inhibition of the ATP and TG-induced $[Ca^{2+}]_{ER}$ reduction. The bar graph shows the mean ± SEM (n = 3) of the $[Ca^{2+}]_{ER}$ taken at 14-min time (black arrow). * $p < 0.05$; ** $p < 0.01$.

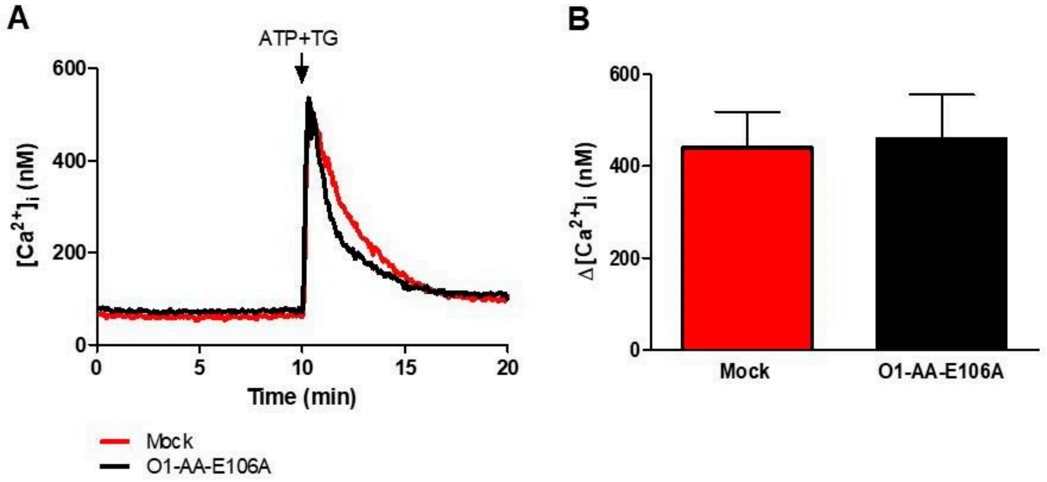


**Figure S3. Cells overexpressing Orai1-S27A/S30A-E106A (O1-AA- E106A) null mutant and cells transfected with mock plasmid displayed the same amplitude in the peak $[Ca^{2+}]_i$ response induced by ATP and TG.** The overexpression of the Orai1-S27A/S30A mutant caused a higher peak $[Ca^{2+}]_i$ response to ATP and TG, as shown in Figure 3 (black bar) of the main text. (**A**) Time course of the $[Ca^{2+}]_i$ response to the addition of 10 μM ATP and 1 μM TG to cells overexpressing O1-AA-E106A null mutant (black trace) or mock cells (red trace). (**B**) Similar average peak $\mu[Ca^{2+}]_i$ response to the addition of ATP and TG between mock cells and those transfected with O1-AA-E106A null mutant. Data showed the mean ± SEM for n = 3.

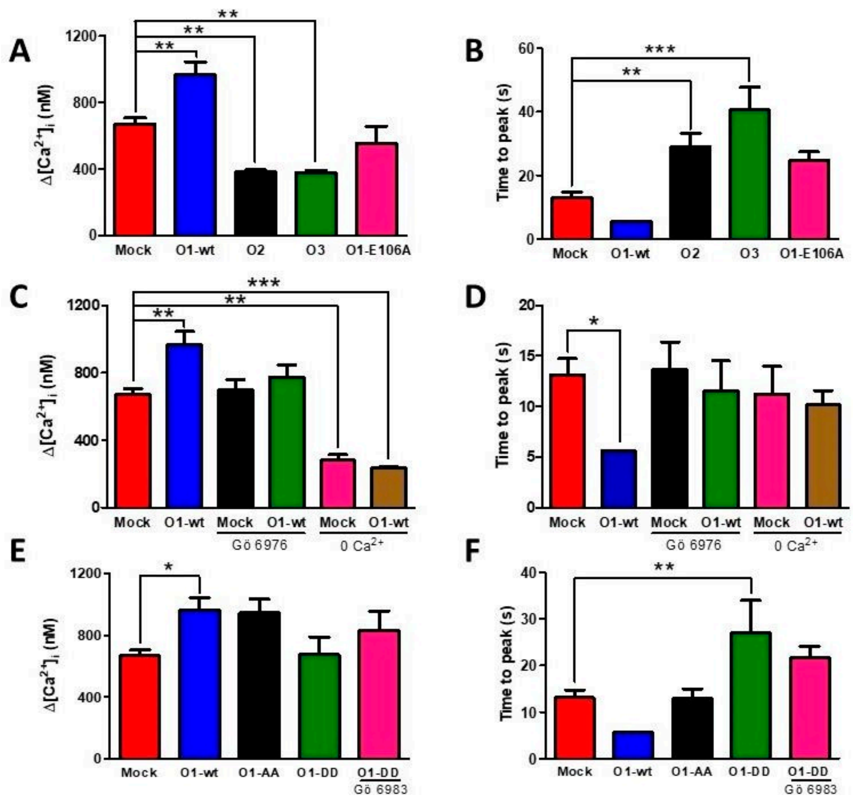


**Figure S4. This figure summarizes data from Figures 1, 2, and 3. Panels show the maximal $[Ca^{2+}]_i$ increase and the time to peak in response to the application of ATP and TG.** (**A**) The expression of the Orai1-wt channel increased the $[Ca^{2+}]_i$ response while (**B**) decreased the time to peak for this response. O2 and O3 channels had the opposite effect compared with O1-wt. This $[Ca^{2+}]_i$ response required a functional O1-wt channel since the dominant-negative null channel O1-E106A did not differ from the mock. The inhibition of PKC with Gö6976 or the absence of external $[Ca^{2+}]$ inhibited (**C**) the increased $[Ca^{2+}]_i$ response induced by ATP and TG and (**D**) the shorter time to peak. The O1 that cannot be phosphorylated or the phosphomimetic channel modified (**E**) the amplitude of the $[Ca^{2+}]_i$ response and also (**F**) the time to peak to the addition of ATP and TG. Data shown are the mean ± standard error of the mean, and the one-way ANOVA with Dunnett's correction was used to determine significant differences to the mock response. * $p < 0.05$, ** $p < 0.01$ and *** $p < 0.001$.

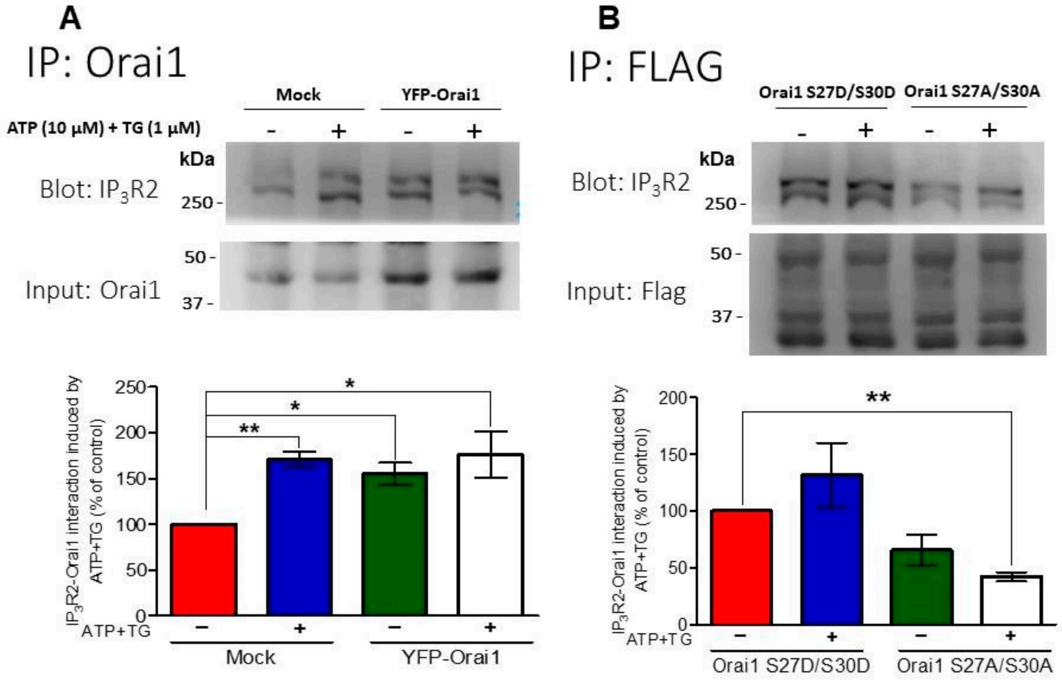


**Figure S5. The Orai1 mutants at S27/S30 amino acid residues affect the interaction with IP3R2 in response to ATP and TG.** (**A**) Immunoprecipitated proteins using an anti-Orai1 antibody from cells transfected with different Orai1 channels. After stimulation with ATP and TG for 3 min, immunoprecipitates from wild-type mutants were blotted with an anti-IP3R2 antibody (upper panel) or anti-Orai1 antibody (lower panel). Western blot of Orai1 channel reflects the input of Orai1 for each condition. The bar graph shows the average O.D. normalized to the mock signal without stimulus. (**B**) Proteins immunoprecipitated with anti-Flag antibody from cells stimulated with ATP and TG for 3 min —to selectively pull down the Orai1 S27D/S30D or Orai1 S27A/S30A mutants— were blotted with anti-IP3R2 antibody (upper panel) or with anti-Flag (lower panel). Notice that ATP and TG decreased the interaction between the IP3R2 and Orai1 S27A/S30A channel, but not with the Orai1 S27D/S30D mutant. The bar graph shows the average O.D. normalized to untreated O1-DD expressing cells signal. All data represented the mean ± SEM. * $p < 0.05$; ** $p < 0.01$ from at least 3 independent experiments.

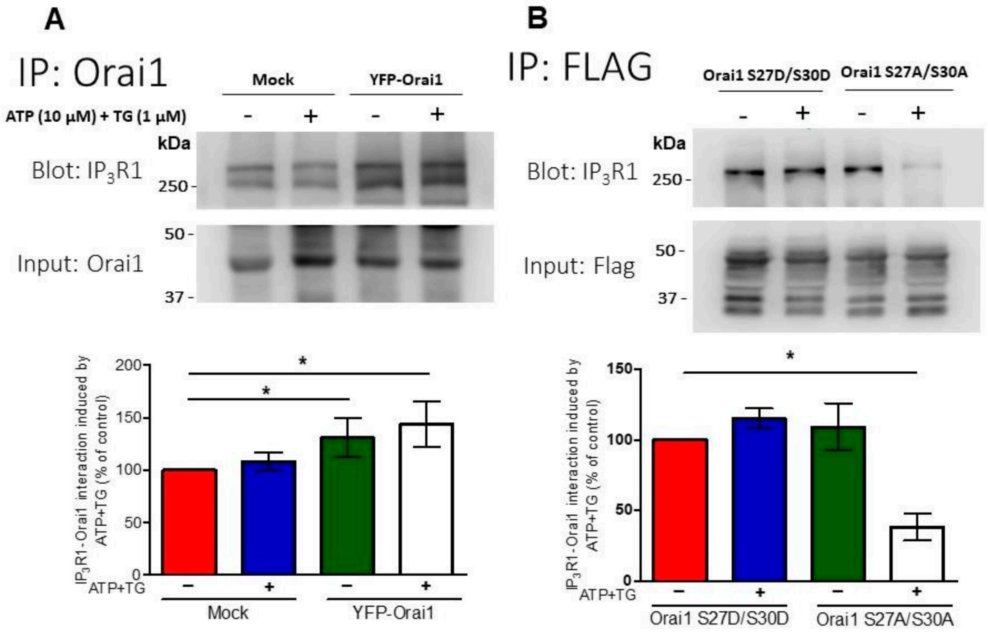


**Figure S6. The Orai1 mutants at S27/S30 amino acid residues affect the interaction with $IP_3R1$ in response to ATP and TG.** (**A**) Immunoprecipitated proteins with an anti-Orai1 antibody from cells transfected with different Orai1 channels, wild type, and mutants, after stimulation with ATP 10 μM and TG 1 μM for 3 min were blotted with an anti-$IP_3R1$ antibody (upper panel) or anti-Orai1 antibody (lower panel). Western blot of Orai1 channel reflects the input of Orai1 involved for each condition. The bar graph shows the average O.D. normalized to the mock signal without stimulus. (**B**) Proteins immunoprecipitated with anti-Flag antibody from cells stimulated with ATP 10 μM and TG 1 μM for 3 min —to selectively pull down the Orai1 S27D/S30D or Orai1 S27A/S30A mutants— were blotted with anti-$IP_3R1$ antibody (upper panel) or with anti-Flag (lower panel). Notice that ATP plus TG strongly decreased the interaction between $IP_3R1$ and the Orai1 S27A/S30A channel. The bar graph shows the average O.D. normalized to untreated O1-DD expressing cells signal. All data represented the mean ± SEM. * $p < 0.05$; from at least 3 independent experiments.

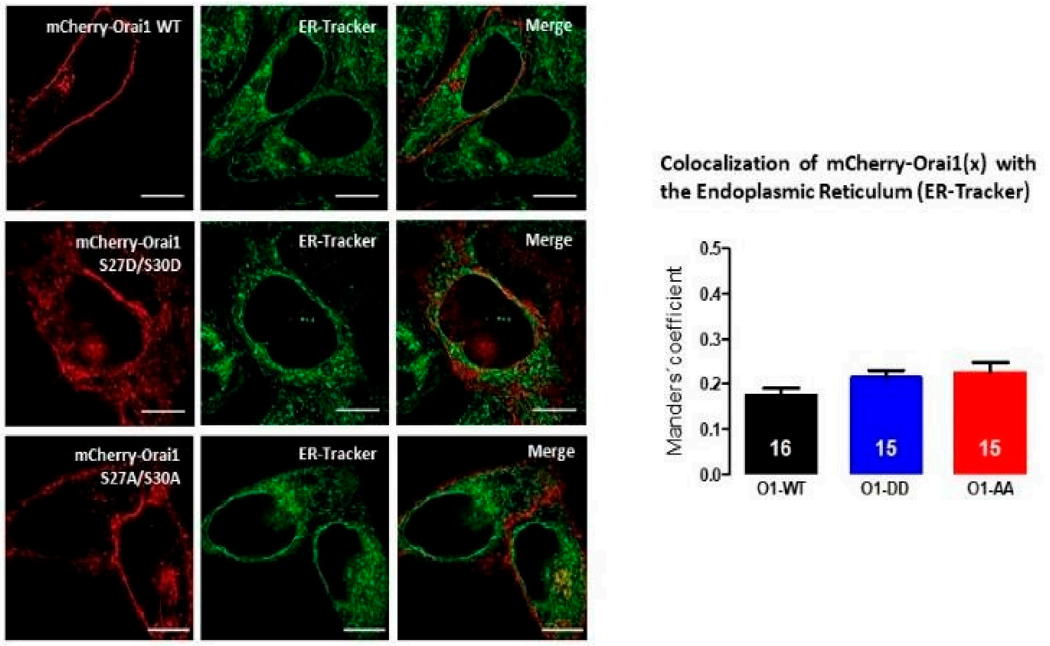


**Figure S7. Colocalization assay of the O1 channels with the endoplasmic reticulum stained with ER-tracker.** Images were acquired as indicated in the methods section. The left panel shows deconvolved confocal images of mCherry-O1-wt, mCherry- O1-DD, and mCherry-O1-AA, while the middle panel shows the same cells co-stained with 1 μM ER-tracker. The right panel shows the merge of the previous panels to determine the degree of colocalization among the different O1 channels and the ER. The Manders' coefficients indicate that, on average, only 20 % of the O1 positive pixels colocalized with ER-tracker regardless of the O1 channel studied. The scale bar indicates 10 μM. The number of cells analyzed is shown within each bar.

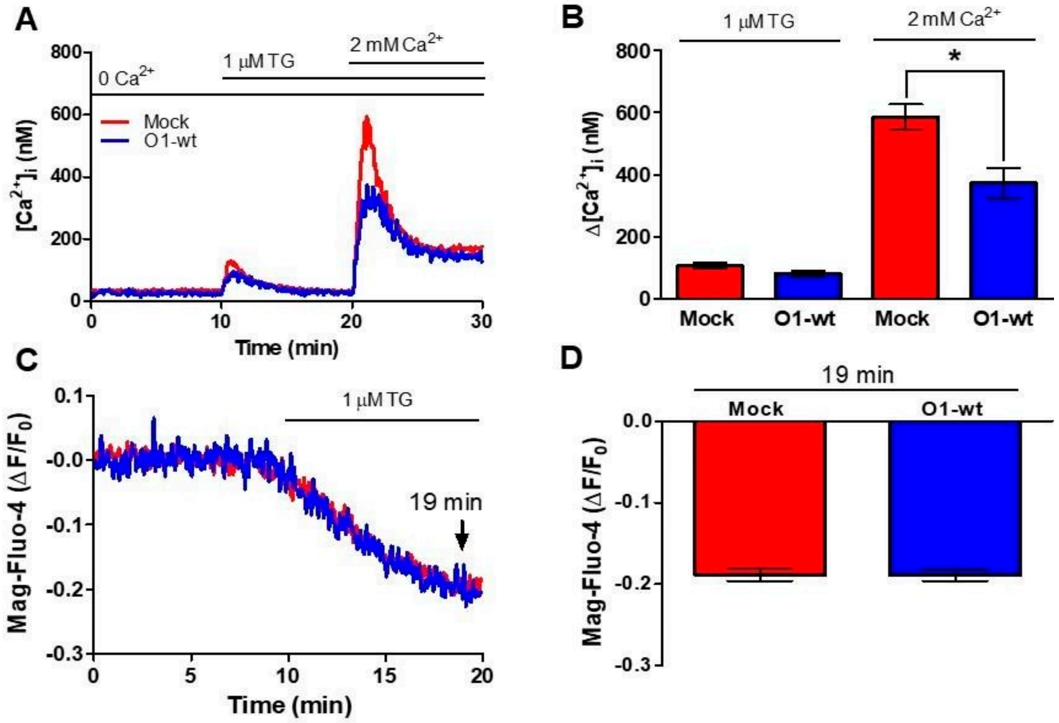


**Figure S8. The overexpression of the O1-wt channel decreased SOCE in HeLa cells.** (**A**) Time course of the fluorescence signal from cells overexpressing Orai1 wild type protein (blue trace) or transfected with a mock plasmid (red trace). The cells loaded with both Fura-2 and Mag-Fluo-4 were placed in a $Ca^{2+}$-free medium (plus 0.1 mM EGTA) and stimulated with TG (1 μM) to induce ER $Ca^{2+}$ depletion followed by $Ca^{2+}$ addition to the medium. (**B**) The bar graph shows the average peak $[Ca^{2+}]_i$ response induced by TG and the peak $[Ca^{2+}]_i$ after $Ca^{2+}$ addition (SOCE), indicating that the expression of the O1-wt channel without STIM inhibited SOCE. (**C**) The time course of the effect of SERCA pump inhibition with TG in a $Ca^{2+}$-free medium was not affected by the presence of the O1-wt channel. (**D**) The bar graph shows the luminal $[Ca^{2+}]$ reduction amplitude at 19 min. All data are represented as mean ± SEM for n = 3. * $p < 0.05$.

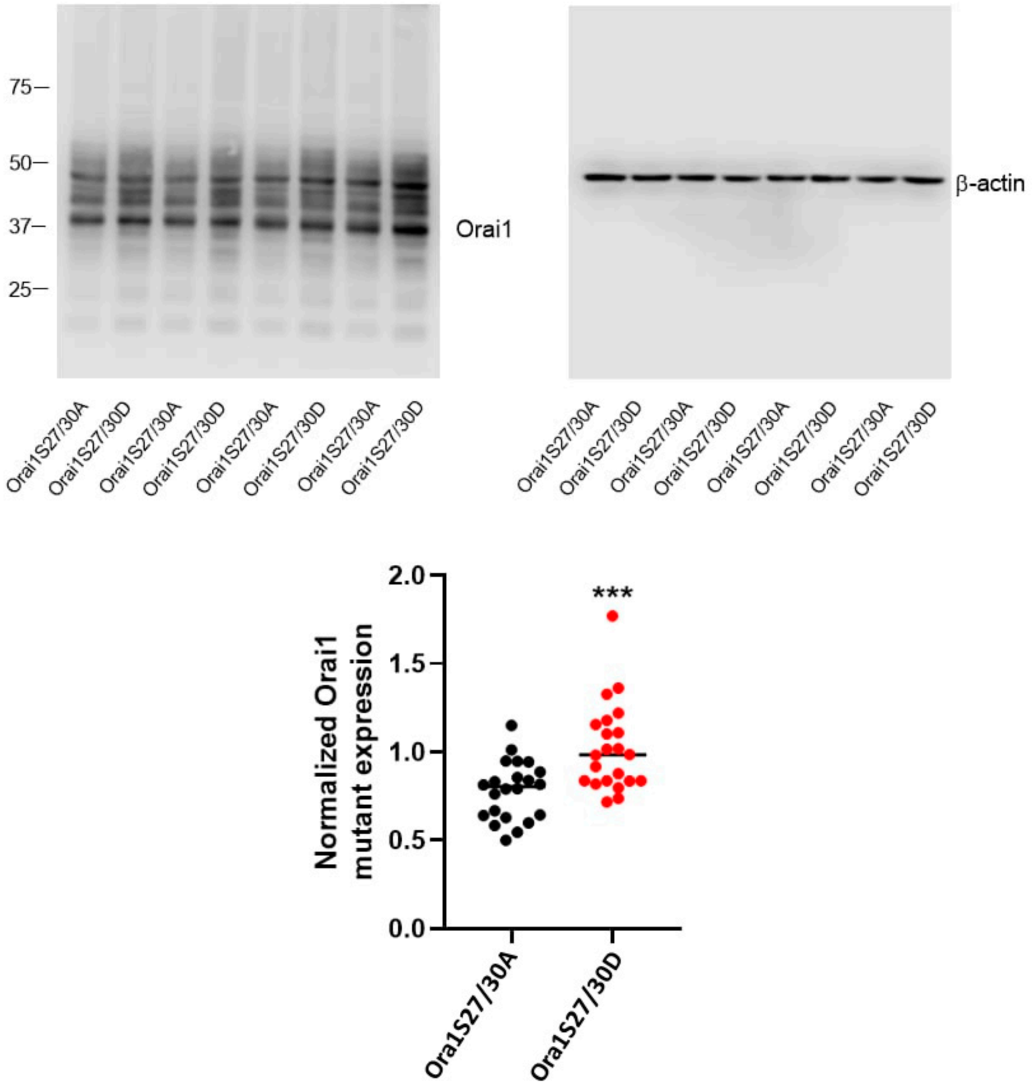


**Figure S9. Analysis of the expression of the Orai1S27/30A and Orai1S27/30D mutants.** HeLa cells overexpressing the double mutant Orai1 S27A/S30A or the phosphomimetic mutant Orai1-S27D/S30D were lysed and subjected to Western blotting with the anti-Orai1 antibody. Membranes were re-probed with an anti-β-actin antibody for protein loading control. Molecular masses indicated on the left were determined using molecular-mass markers run in the same gel. Blots are representative of six separate experiments. The scatter plot represents the quantification of the Orai1 expression. Data were statistically analyzed using Student´s *t*-test (*** $p < 0.001$, compared to Orai1 expression after transfection with the Orai1S27/30A).
